# Reconfigurable Multifunctional Metasurfaces for Full-Space Electromagnetic Wave Front Control

**DOI:** 10.3390/mi15111282

**Published:** 2024-10-22

**Authors:** Shunlan Zhang, Weiping Cao, Jiao Wang, Tiesheng Wu, Yiying Wang, Yanxia Wang, Dongsheng Zhou

**Affiliations:** 1 School of Information and Communication, Guilin University of Electronic Technology, Guilin 541004, China; 2 Hebei Jinghe Electronic Technology Incorporated Company, Shijiazhuang 050200, China

**Keywords:** metasurface, multifunctional, reconfigurable, transmission-reflection integrated, full-space

## Abstract

In order to implement multiple electromagnetic (EM) wave front control, a reconfigurable multifunctional metasurface (RMM) has been investigated in this paper. It can meet the requirements for 6G communication systems. Considering the full-space working modes simultaneously, both reflection and transmission modes, the flexible transmission-reflection-integrated RMM with p-i-n diodes and anisotropic structures is proposed. By introducing a 45°-inclined H-shaped AS and grating-like micro-structure, the polarization conversion of linear to circular polarization (LP-to-CP) is achieved with good angular stability, in the transmission mode from top to bottom. Meanwhile, reflection beam patterns can be tuned by switching four p-i-n diodes to achieve a 1-bit reflection phase, which are embedded in the bottom of unit cells. To demonstrate the multiple reconfigurable abilities of RMMs to regulate EM waves, the RMMs working in polarization conversion mode, transmitted mode, reflected mode, and transmission-reflection-integrated mode are designed and simulated. Furthermore, by encoding two proper reflection sequences with 13×13 elements, reflection beam patterns with two beams and four beams can be achieved, respectively. The simulation results are consistent with the theoretical method. The suggested metasurface is helpful for radar and wireless communications because of its compact size, simple construction, angular stability, and multi-functionality.

## 1. Introduction

A metasurface (MS) is a surface with periodic or aperiodic structures, consisting of subwavelength elements, which possess the unique ability to control the amplitude [1,2], phase [2,3], and polarization states [4,5] of incident electromagnetic (EM) waves. They have some advantages such as a lower profile, low insertion loss, and easy integration with other circuits [6,7]. With the help of innovative techniques for modulating electromagnetic waves and a variety of useful applications, including diffusion, anomalous reflection and refraction [8,9], radar cross-section reduction [10,11], beam scanning [12,13,14], focusing [15,16], polarization conversion [17,18,19], and holography [20], researchers have flexibly designed MSs based on the generalized Snell’s law [21]. Nonetheless, passive unit cells make up the majority of these designs. Their functions are set once they are produced, and they can only be used for a limited number of pre-planned uses. As a result, they are unable to meet the growing needs for communications and multifunctional devices.

Compared with passive MSs whose functions are fixed [22], reconfigurable MSs possess a stronger superiority [23,24]. Versatile features are expected because of their dynamic status changes. Therefore, the reconfigurable multifunctional metasurfaces (RMMs) can be controlled by electrical, optical, mechanical, and thermal means, which have been developed. These RMMs control EM waves from microwave to terahertz bands; furthermore, they are useful in a variety of applications, including antenna design [25], polarization conversion [26,27,28,29], and beam steering [30,31,32]. In the meantime, tunable MSs have been designed using functional materials such as liquid crystals [33], graphene [34,35], and vanadium dioxide (VO2) [36]. For example, using the insulator-to-metal transition feature of VO2, the researchers in [36] created a switchable MS that can accomplish broadband absorption and reflection. However, these RMMs mostly operate in either the transmission or reflection mode to regulate EM waves in half-space, and the other space is unutilized. Nonetheless, RMMs in full-space will possess a wider prospect, particularly with the growing demands for highly integrated and more powerful devices in 6G communication systems.

Driven by the imperative demand for both design integration and miniaturization for 6G applications, based on the earlier research in [37], we further present a flexible transmission-reflection-integrated RMM with p-i-n diodes and an anisotropic structure (AS), which can convert the *x*-polarized EM waves to circular polarization (CP) in the transmission mode from top to bottom, and control reflection beam patterns of the y-polarized EM waves in the reflection mode from bottom to top. The suggested unit cell for the MS is made up of five delicately designed metal patterns separated by four substrate layers. The upper three metal patterns complete the polarization conversion of linear-to-right hand circular polarization (LP-to-CP). By integrating four p-i-n diodes into metal layer 5, 1-bit tunable reflection phases are realized, which can control the y-polarized EM wave reflection patterns from bottom to top. To verify its adjustable property, a 13 × 13 array is designed, which can reflect beam patterns with two beams and four beams in reflection mode through the modulation of the switchable status on p-i-n diodes. Unlike previous research, our designs provide a feasible way of realizing adjustable multifunctional MSs operating in full space, which possess good angular stability and can result in many fascinating applications in wireless communications and radar.

## 2. Metasurface Unit Cell Design

**Figure 1:**
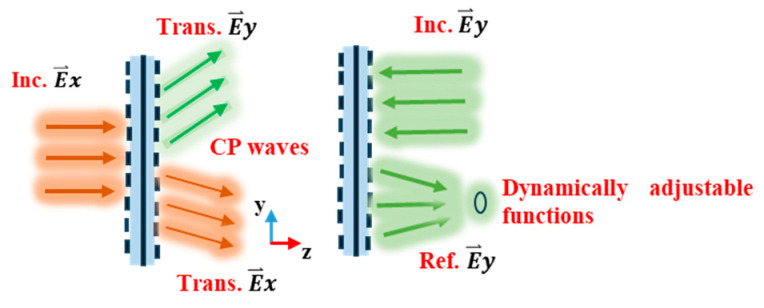
The schematic diagram of the suggested RMM working for full-space.

**Figure 2:**
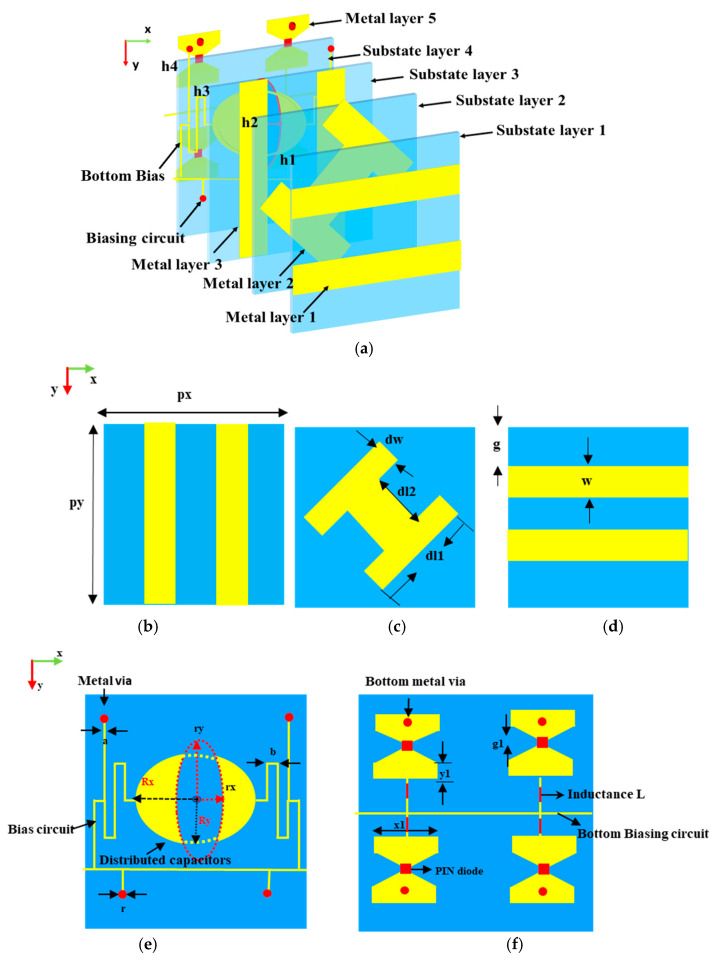
The RMM unit cell structure. (**a**) Schematic of the unit cell; (**b**–**f**) structures of five metal patterns from 1 to 5.

Through rigorous structural design, numerous functionalities can be extracted from a single geometric structure, evoking unique reactions, as described in Figure 1. The RMM unit cell structure is depicted in Figure 2, where five metal layers, divided by four substrates, make up the RMM unit cell. Metal layers 1 to 3 are etched onto the Arlon AD255A (tm) substrate with $\varepsilon_{r}=2$.55 and tanδ=0.0015. Additionally, the F4B substrate is etched with the patterns of metal layers 4 and 5, which exhibit $\varepsilon_{r}=2$.65 and tanδ=0.0015. In order to produce orthogonally polarized waves when the incoming waves are linearly polarized, the element structure must be asymmetric along the incident wave’s polarized direction. Meanwhile, the structure needs to be symmetrical in the direction along a 45° angle, which can enhance the polarization conversion ratio [38]. As described in Figure 2c, the pattern of metal layer 2 is a 45°-inclined H-shape, which completes the polarization conversion of LP-to-CP. As depicted in Figure 2b,d, the polarization grids on metal layers 1 and 3 are along the *y*- and *x*-directions, respectively, which improve the conversion attributes of the *x*-polarized EM waves and enhance polarization conversion purity. In conjunction with the metal grating on metal layer 3, four open trapezoid patches on metal layer 5 work as an artificial magnetic conductor (AMC). The two dc bias signal lines are designed on metal layers 4 and 5, as presented in Figure 2e,f. To isolate the high-frequency signals, two crescent-distributed capacitors and symmetrically distributed inductances are integrated into the bias layer of metal layer 4. In the meantime, the bias layer on metal layer 5 adopts the four inductors with *L* = 270 nH to choke RF currents. Through four metallized via-holes, the bottom trapezoid patches and the bias layer of metal layer 4 are connected. Four red square components on metal layer 5 are p-i-n diodes, which connect with a pair of symmetric trapezoids, respectively, as shown in Figure 2f. The proposed RMM is simulated and analyzed using the software Ansys HFSS 2018, the Floquet port, and periodic boundary conditions. The unit designated in Figure 2 has its optimal parameters shown in Table 1.

**Figure 3:**
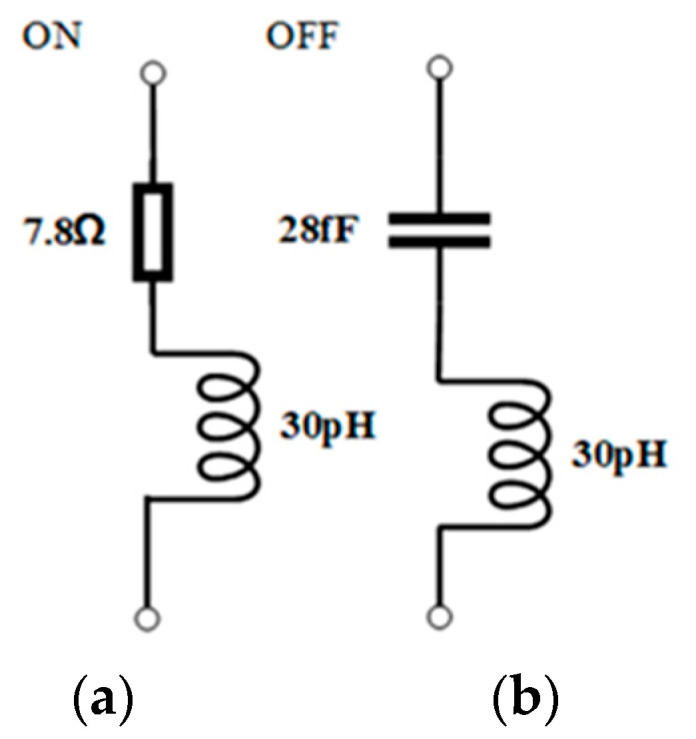
Analogous circuit of p-i-n diodes in different states: (**a**) ON state, (**b**) OFF state.

The p-i-n diode used is MADP-000907-14020 from MACOM, Lowell, Massachusetts, USA. According to the data sheet, the forward-biased diode can be analogous to a series RL circuit with resistance R=7.8 Ω and inductance L = 30 pH, as described in Figure 3a. Meanwhile, the reverse-biased diode can be analogous to a series LC circuit with resistance L = 30 pH and capacitance *C* = 28 fF, as depicted in Figure 3b.

Analysis of the RMM in transmitted and reflected modes is carried out independently. The parts below provide a thorough analysis.

## 3. Transmission Mode

When the *x*-polarized incoming waves are impinged on the RMM from top to bottom, the transmitted wave will be converted in polarization due to matching electric fields being excited. In the meantime, the RMM operates in transmission mode, and works as a polarizer.

### 3.1. Working Principle

**Figure 4:**
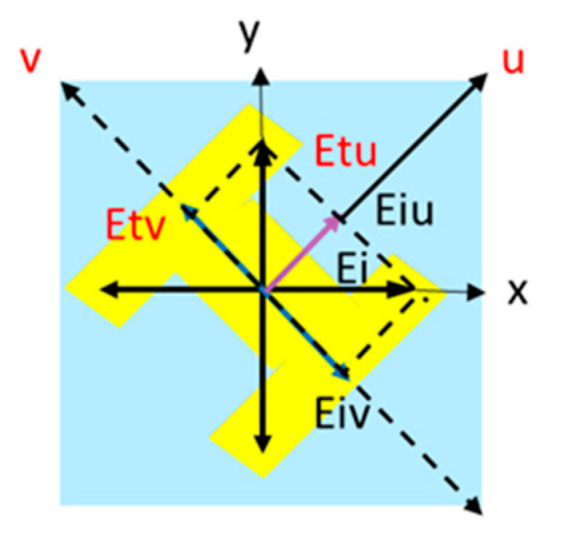
The electric field along the x-axis splitting into the u and v components, where the right-hand coordinate system is assumed, and the z axis is directed towards the reader.

To realize the polarized conversion of LP-to-CP with an *x*-polarized incoming wave impinging on the RMM, the element structure should be asymmetric along the *x*-direction and embrace a 45°-inclined H-shape, which can translate the *x*-polarized wave into two perpendicularly polarized waves and produce different accumulations of phases. As depicted in Figure 4, the *x*-polarized incoming wave ${\overset{⃑}{E}}_{i}$ can be divided into the tangential (*u*) and normal (*v*) directions at the center point of the inclined H-shape, and can be written as

(1) $${\overset{⃑}{E}}_{i}=\hat{x}E_{ix}e^{-jkz}=\hat{u}E_{iu}e^{-jkz}+\hat{v}E_{iv}e^{-jkz}$$

where $E_{iu}=E_{iv}=E_{ix}/\sqrt{2}$; k is the wavenumber; and $\hat{x},\hat{u}$, and $\hat{\nu }$ are the unit vectors with respect to the *x*-axis and *u*- and *v*-directions, respectively. The transmitted fields can be written using

(2) $${\overset{⃑}{E}}_{t}=\left[\hat{u}\left(T_{uu}E_{iu}+T_{uv}E_{iv}\right)+\hat{v}\left(T_{vu}E_{iu}+T_{vv}E_{iv}\right)\right]e^{-jkz}$$

where $T_{uu}=\left|T_{uu}\right|e^{j\varphi_{uu}},T_{vu}=\left|T_{vu}\right|e^{j\varphi_{vu}},T_{uv}=\left|T_{uv}\right|e^{j\varphi_{uv}}$, and $T_{vv}=\left|T_{vv}\right|e^{j\varphi_{vv}}$ are transmission coefficients.

When $\left|T_{vu}\right|=\left|T_{uv}\right|=0,\left|T_{uu}\right|=\left|T_{vv}\right|$, and $\Delta \varphi =\varphi_{uu}-\varphi_{vv}=\pi$,

(3) $${\overset{⃑}{E}}_{t}=\left(\hat{v}-\hat{u}\right)T_{vv}\left(E_{ix}/\sqrt{2}\right)e^{-jkz}=\hat{y}E_{ty}e^{-jkz}$$

which implies that the EM waves are converted from the *x*-polarized waves to the *y*-polarized ones. When the phase differences $\Delta \varphi =\varphi_{uu}-\varphi_{vv}=0$, the polarization of the transmission wave is still along the *x*-direction. If Δφ=±90°, the incoming linearly polarized waves are transformed into circularly polarized waves, which can be described as follows:

(4) $$\begin{array}{l} {\overset{⃑}{E}}_{t}=\left(\hat{x}E_{tx}+\hat{y}E_{ty}\right)e^{-jkz}\\ \;=\left(\hat{x}\left|T_{xx}\right|e^{j\varphi_{xx}}+\hat{y}\left|T_{yx}\right|e^{j\varphi_{xx}\pm \pi /2}\right)E_{ix}e^{-jkz} \end{array}$$

where $T_{xx}=\left|E_{tx}/E_{ix}\right|e^{j\varphi_{xx}}\;and{\;T}_{yx}=\left|E_{ty}/E_{ix}\right|e^{j\left(\varphi_{xx}\pm \pi /2\right)}$ are transmission coefficients of co- and cross-polarized components under the *x*-polarized incident wave. It is clear from Equation (4) that the phase differences Δφ and the co-polarized and cross-polarized transmission coefficients ($T_{xx}$ and $T_{yx}$*)* can be used to identify the state of polarization for the transmitted field. When $\left|T_{xx}\right|=\left|T_{yx}\right|,if$ Δφ ≈ 90° and Δφ ≈−90°, a polarized conversion of LP-to-LHCP and LP-to-RHCP is achieved, respectively, whereas when $\left|T_{xx}\right|\neq \left|T_{yy}\right|$, an elliptic polarization wave is generated.

From Equation (4), it is concluded that controlling polarization can be accomplished through altering Δφ, which is mostly decided by the structure of resonant unit cells. Δφ is a function of frequency when metasurfaces are unreconfigurable structures, meaning that distinct polarized conversion functions will be generated at distinct frequencies [37].

### 3.2. Simulation Results

We further discuss circular polarization conversion through the axial ratio (*AR*). The transmission coefficients of two waves with orthogonal polarization under an *x*-polarized incoming wave can be used to determine the *AR* parameter, which is crucial for assessing the electromagnetic waves’ level of circular polarization.

(5) $$AR=\frac{\sqrt{\left[T_{xx}^{2}+T_{yx}^{2}+\sqrt{T_{xx}^{4}+T_{yx}^{4}+2{\left(T_{xx}T_{yx}\right)}^{2}cos(2\Delta \varphi )}\right]}}{\sqrt{\left[T_{xx}^{2}+T_{yx}^{2}-\sqrt{T_{xx}^{4}+T_{yx}^{4}+2{\left(T_{xx}T_{yx}\right)}^{2}cos(2\Delta \varphi )}\right]}}$$

From the previous analysis, we are aware that in order to produce circularly polarized waves, the two orthogonal components of the transmitted electric field must have a phase difference of Δ*φ* = ±90° and equal amplitude, i.e., $\left|T_{xx}\right|=\left|T_{yx}\right|$. If the *AR* is less than 3 dB, it is roughly circular polarization in practice.

**Figure 5:**
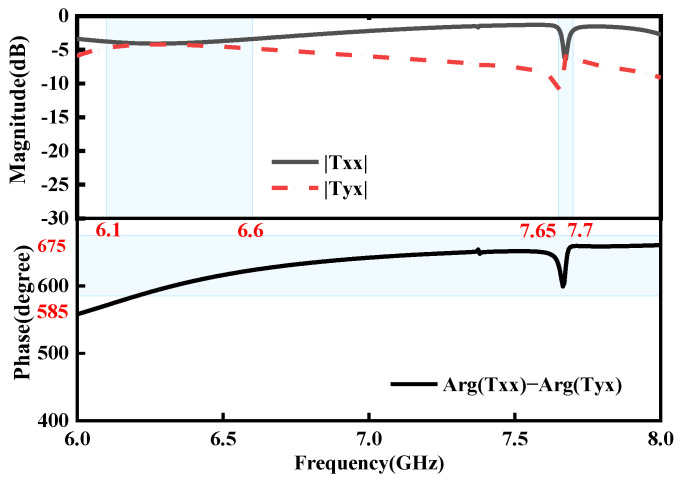
Simulated co-polarized and cross-polarized transmission magnitudes and phase differences under the *x*-polarized EM wave in the ON state.

**Figure 6:**
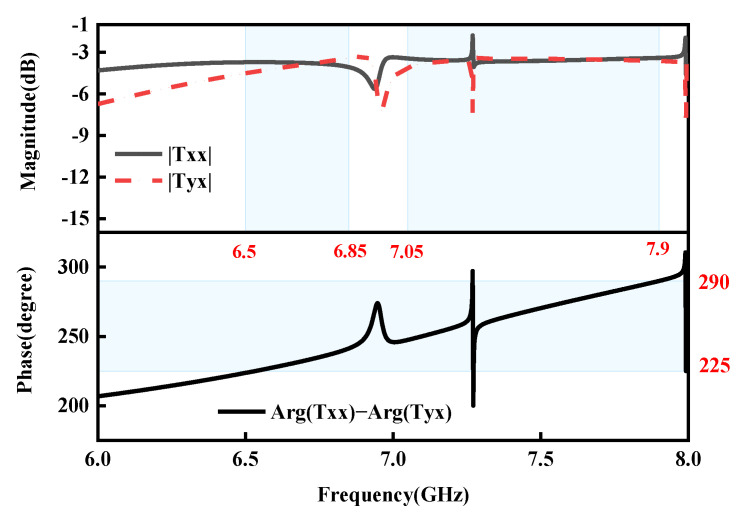
Simulated co-polarized and cross-polarized transmission magnitudes and phase differences under the *x*-polarized EM wave in the OFF state.

The full-wave simulations are used to examine the RMM’s transmission magnitudes and phase discrepancies in order to verify our design. In Figure 5, the simulated co-polarized and cross-polarized transmission magnitudes and phase differences are displayed when the diode is forward-biased. It is evident that ${|T}_{xx}|$ and $\left|T_{yx}\right|$ are over −10 dB and nearly equal within the working frequency ranges of 7.65–7.7 GHz and 6.1–6.6 GHz, and that the phase differences are close to 270°, which indicates the transmission of RHCP waves. When the diode is reversebiased, Figure 6 displays the simulated co-polarized and cross-polarized transmission magnitudes and phase discrepancies. Thus, an excellent circularly polarized transmission wave is achieved in the two bands of 6.5–6.6 GHz and 7.65–7.7 GHz in both the ON and OFF states.

**Figure 7:**
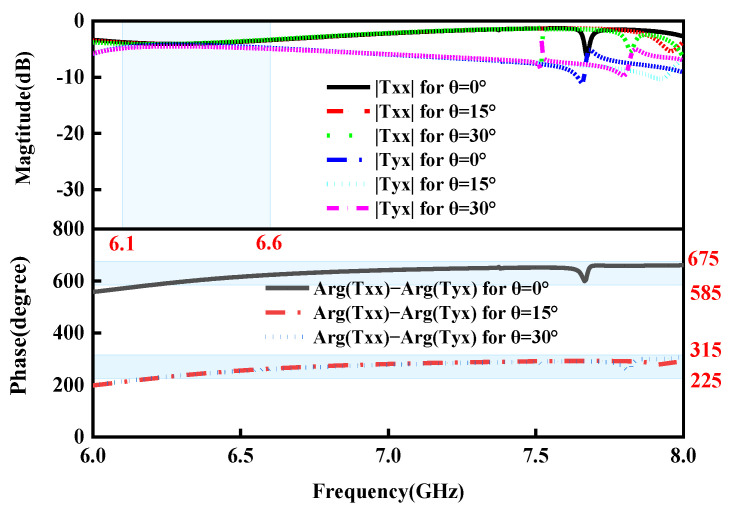
Simulated co- and cross-polarization transmission magnitudes and phase differences for different incident angles under the *x*-polarized EM waves in the ON state.

**Figure 8:**
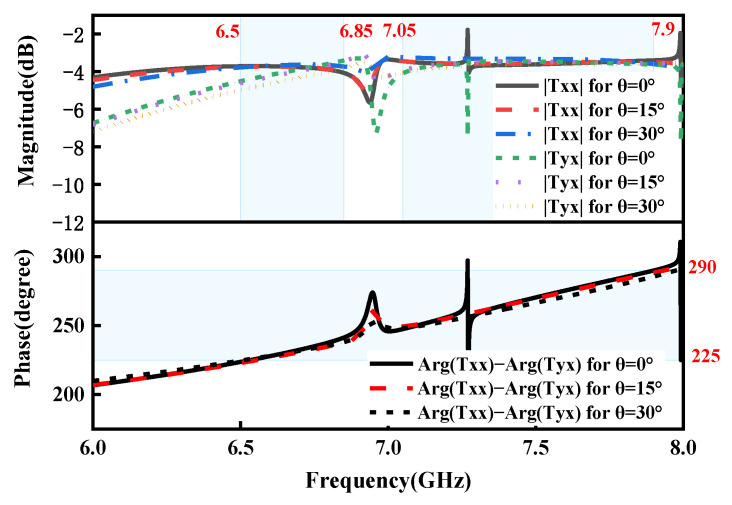
Simulated co- and cross-polarization transmission magnitudes and phase differences for different incident angles under the *x*-polarized EM waves in the OFF state.

In order to look into the impact of oblique incidence on transmission performance, the variation in transmission performances is investigated for different obliques under the *x*-polarized EM waves from top to bottom. Figure 7 and Figure 8 present the co- and cross-polarized transmitted magnitudes and phase differences versus operating frequency at oblique incidence with the incidence angles θ=0°, 15°, and 30° in the ON and OFF states, respectively. It is obvious from Figure 7 and Figure 8 that the curved lines of the simulated co- and cross-polarization transmission magnitudes and phase differences are nearly coincident with those under normal incidence in the ON and OFF states, respectively, which shows the great angular stability in transmission mode in the ON and OFF states.

## 4. Reflection Mode

When the y-polarization incident wave is impinged on the RMM from bottom to top, by switching the p-i-n states and designing the discontinuous reflection phases, the wavefront of EM waves can be freely tailored, opening a wide range of new phenomena and applications, including beam patterns with two beams and four beams. Meanwhile, the RMM works in reflection mode and acts as a reflector.

### 4.1. Performance Analysis

**Figure 9:**
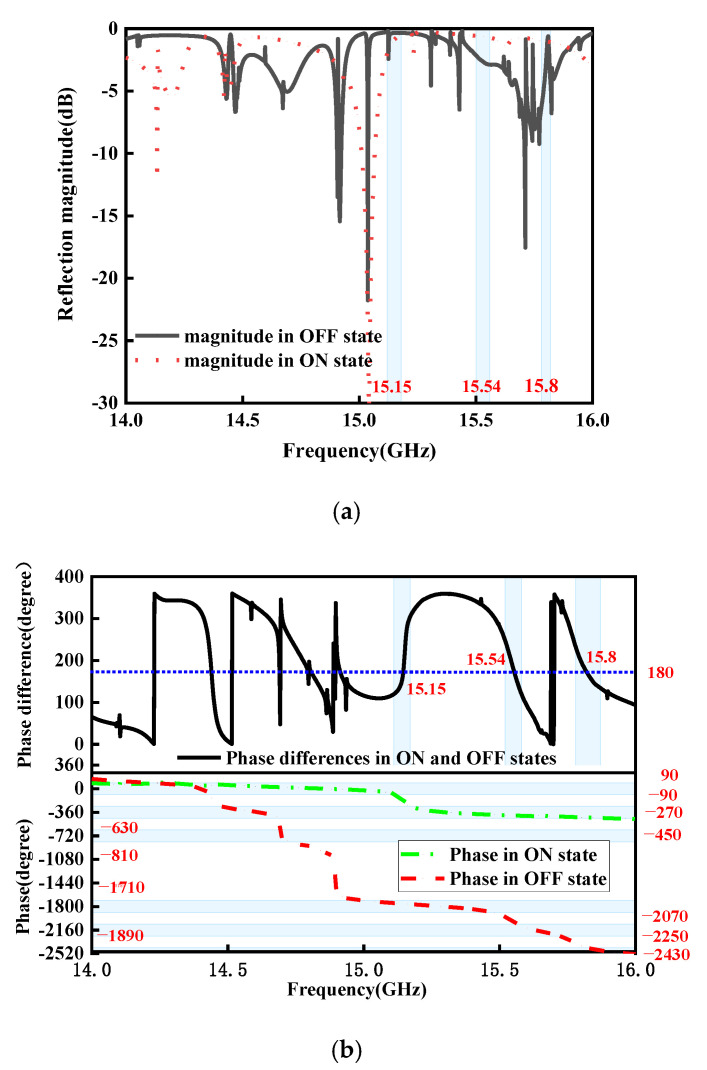
Simulated reflection coefficients of the presented MS in the ON and OFF states: (**a**) reflection coefficient; (**b**) reflection phase and reflection phase differences between p-i-n diode ON and OFF states.

According to the analysis above, we can determine that four open trapezoidal patches on metal layer 5 serve as an AMC in conjunction with the metal grating on metal layer 3, which acts as the ground of the AMC. To verify this, the simulated reflection performances are analyzed. An AMC can be generated by impinging y-polarized EM waves on the MS from bottom to top almost in the frequency range of 14–16 GHz, which can be used for stealth materials in radars [39] or high-gain antennas [40]. Figure 9 presents reflection coefficients, phases, and phase differences of the proposed flexible regulated MS under the y-polarized normal incidence in the ON and OFF states within a bandwidth of 14–16 GHz. As shown in Figure 9a, folded reflected phases are almost in the range of −90°~+90°, and most of the incident waves are reflected with reflected coefficients (${|R}_{yy}|=\left|E_{ry}/E_{iy}\right|$) greater than −10 dB in each state in the operating band, which indicates that the proposed MS works as an AMC. Furthermore, it is obvious from Figure 9b that the reflection phases are discernibly distinct in every state, and the phase discrepancies between adjacent states fall in the range of (180° − 10°, 180° + 10°) around 15.15 GHz, 15.54 GHz, and 15.8 GHz, by switching simultaneously the four p-i-n diodes on metal layer 5 to operate in the ON/OFF states. Therefore, 1-bit coding elements can be obtained at 15.15 GHz, 15.54 GHz, and 15.8 GHz, meaning that the gradient reflection phase distribution of θ and θ+180° can be produced by the suggested metasurface working from state 1 to state 2. As a result, the element configuration in each state can be thought of as a fundamental digital element. Two different element configurations yield 1 bit, simulating the state 1 and state 0 units, respectively.

**Figure 10:**
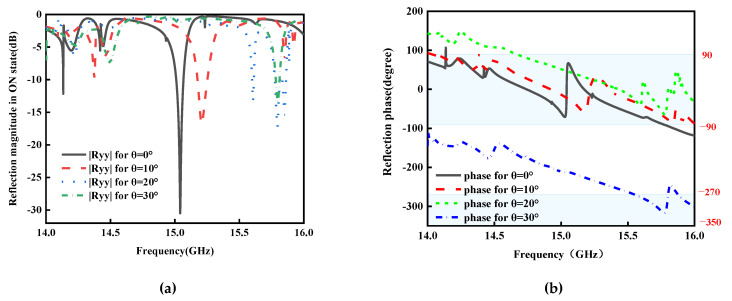
Simulated reflection magnitudes and phases for different incident angles θ in the ON states under the y-polarized wave from bottom to top: (**a**) magnitudes, (**b**) phases.

**Figure 11:**
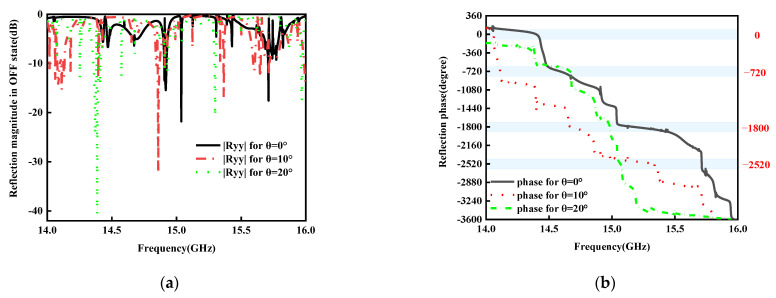
Simulated reflection magnitudes and phases for different incident angles θ in the OFF states under the y-polarized wave from bottom to top: (**a**) magnitudes, (**b**) phases.

Subsequently, the performance of reflection is analyzed for various oblique incidences in reflection modes. The reflection magnitudes and phases versus operating frequency with variable incident angles θ in the ON and OFF states are shown in Figure 10 and Figure 11. From these figures, it is concluded that the reflection performance is almost stable up to 30° in the ON state and 20° in the OFF state in the band of 14–16 GHz, which implies that the proposed RMM possesses better stability at oblique incidence in the reflection mode. The above findings have conclusively demonstrated that the suggested RMM can still operate as a digital coding MS at 20 degrees of oblique illumination in each state. Greater incident angles cause the EM wave to take an extra path between the ground (metal layer 3) and the bottom, which increases the phase difference, resulting in destructive interference [38],

(6) $$\Delta \phi =\phi_{oblique}-\phi_{normal}=2\sqrt{\varepsilon_{r}}kd\left(\frac{1}{\sqrt{1-\frac{\sin^{2}\theta }{\varepsilon_{r}}}}-1\right)$$

where θ represents the incident angle with respect to normal incidence, k signifies the vector of wave propagation in free space, d indicates the thickness of the substrate, and $\varepsilon_{r}=2.65$. Reflection performance degrades severely for incidence angles more than 30° in the ON state and 20° in the OFF state. Folded reflection phases are out of −90°–90°. It is obvious from Equation (6) that higher-order modes or grating lobes are the cause of the performance deterioration. It can be concluded from the above analysis that the overall trend in the reflection performance under oblique incidence also meets the majority of large-angle incidence design criteria despite not being as stable as the transmission mode.

### 4.2. Reconfigurable Reflection Coding MSs

These coding particles could be arranged to form a coding metasurface with various encoding sequences in two dimensions, which possesses different reflection patterns. According to array theory, the radiation pattern of a given encoding sequence, which is made up of N×N equal-sized unit cells with dimension *D*, can be analytically calculated. In the far-field area, the specific characteristic of each coding particle becomes hazy because of the subwavelength characteristic of the digital particle with a reflection phase of φ(m,n) (either θ or θ+180° in the 1-bit case) for the mnth element. The far-field-function-reflected metasurface at the normal incidence of plane waves is written as

(7) $$\begin{array}{l} f(\theta ,\varphi )=f_{e}(\theta ,\varphi )\\ \sum_{n=1}^{N}\;\exp\left\{-i\{\varphi (m,n)+kDsin\theta [(m-1/2)cos\varphi +(n-1/2)sin\varphi ]\}\right\} \end{array}$$

where the lattice pattern function is denoted by $f_{e}(\theta ,\varphi )$ and the elevation and azimuth angles are represented by θ and φ, respectively. The relative phase of the “0” element has been assumed to be zero for simplicity’s sake, and the term $f_{e}(\theta ,\varphi )$ in Equation (7) has been neglected because it becomes ambiguous in the far-field. Using any given encoding sequence, we may determine the directivity function $Dir\left(\theta ,\varphi \right)$ from Equation (7), which can be represented as

(8) $$Dir\left(\theta ,\varphi \right)=\frac{4\pi {\left|f\left(\theta ,\varphi \right)\right|}^{2}}{\int_{0}^{2\pi }\int_{0}^{\pi /2}{\left|f\left(\theta ,\varphi \right)\right|}^{2}sin\theta d\theta d\varphi }$$

**Figure 12:**
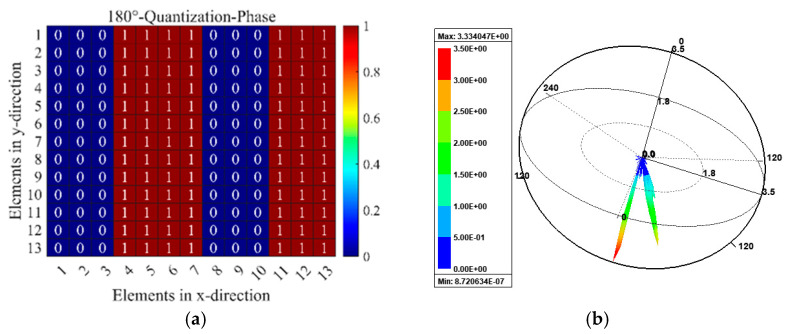
Schematic illustration of 0001111…… coding MSs under the y-normal illumination from bottom to top with dual-beam pattern: (**a**) coding phase profile, (**b**) simulated 3D far-field patterns.

**Figure 13:**
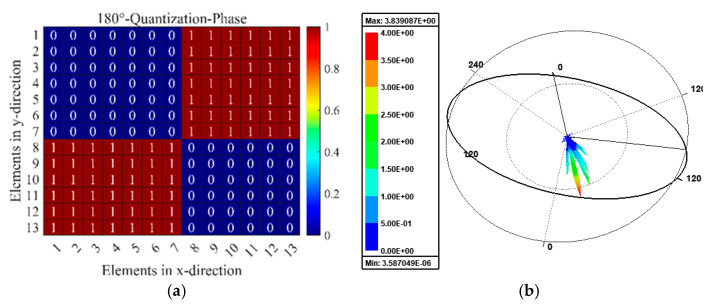
Schematic illustration of chess-board periodic coding metasurface under the y-normal illumination with quad-beam pattern: (**a**) coding phase profile, (**b**) simulated 3D far-field patterns.

In order to investigate the aforementioned beam modulation performance, the 1-bit digital MS is designed, which consists of 13 × 13 unit cells in the total size of 234 mm × 234 mm. The various reflection fields will be produced by various coding sequences in the reflection mode, and each particle can be independently controlled. Full-wave numerical simulations are used to finish all numerical simulations. Figure 12 presents the reflection pattern of the proposed RMM with the 0001111000111 coding sequence, under the y-normal illumination from bottom to top. The coding sequence is shown in Figure 12a. From Figure 12b, it is evident that normal-incidence plane waves are redirected in two symmetrical directions, which is consistent with the theoretical predictions in Equation (7). Figure 13 depicts the reflection pattern of the chess-board periodic coding MS under the y-normal illumination from bottom to top. The chess-board coding sequence with 01/10 is shown in Figure 13a. From Figure 13b, it can be obviously found that the normal-incidence plane waves are scattered in four symmetrical directions, which further verifies the theory derivation results in Equation (7). The comparison of the designed RMM with some existing multifunction MSs for full space are listed in Table 2. It can be observed that the proposed RMM possesses real-time multifunctional adjustability and a low profile. Further, transmission and reflection of the designed RMM have good angular stability compared to other existing multiple MSs.

## 5. Conclusions

In this paper, an important design has been implemented: a flexible transmission-reflection-integrated RMM with p-i-n diodes and an AS, which can realize the EM wave polarization conversion from the LP to CP in the transmission mode. As a good structure from top to bottom, it can modulate reflection beam patterns by switching the p-i-n diodes embedded into metal 5. Taking the transmitted mode as an example, the physical mechanism of multifunctionalities was developed. An RMM with 13×13 elements was designed and simulated, which can scatter reflection beam patterns with two beams and four beams by switching p-i-n diodes. The efficiency of the suggested design is confirmed by the simulation results, which agree with the theoretical predictions. Our design provides a new method for building an RMM in full space and possesses enormous potential applications in radar and wireless communications.

## Figures and Tables

**Table 1:** Dimensions of the proposed unit cell (unit: mm).

Parameter	px	py	g	w	g1	x1	y1
Value	18	18	4.05	1.35	0.33	5.5	1
Parameter	h1	h2	h3	h4	dl1	dl2	dw
Value	3.175	0.813	0.254	1.524	9	9	2.7
Parameter	a	b	r	Rx	rx	Ry	ry
Value	0.1	1	0.1	4.2	1.66	2.5	3.46

**Table 2:** Comparison of the proposed RMM with some recently reported full-space MSs.

Refs.	Real-Time Tunable Phase	Active Element	Layer Number	Bias Circuit Design	Profile
[41]	Yes	P-i-n	9	No	High
[42]	No	-	5	-	Low
[43]	Yes	P-i-n	11	Yes	Low
[44,45]	No	-	7	-	Low
This work	Yes	P-i-n	9	Yes	Low

## Data Availability

The original contributions presented in the study are included in the article, further inquiries can be directed to the corresponding author.
